# Primary Breast Carcinoma in Ectopic Breast Tissue in the Suprapubic Region Presenting as a Subcutaneous Nodule: A Case Report and Review of the Literature

**DOI:** 10.7759/cureus.89015

**Published:** 2025-07-29

**Authors:** Diana C Correa Sandoval, Diego A Guajardo Nieto, Jose L Guzman Murguia

**Affiliations:** 1 Center for Breast Care, Hospital Angeles Valle Oriente, San Pedro Garza Garcia, MEX

**Keywords:** breast carcinoma, diagnosis, differential diagnosis of breast cancer, ectopic tissue, general surgery and breast cancer

## Abstract

Ectopic breast tissue (EBT) is an uncommon congenital condition resulting from incomplete involution of the milk line. Primary carcinomas arising in EBT have been reported in a small number of cases, most frequently in the axilla, but they may occur anywhere along the mammary line. Here, we present the case of a 69-year-old woman who presented with an asymptomatic subcutaneous nodule located at the end of a Pfanneistein’s laparotomy scar in the suprapubic region. Histopathological examination revealed invasive ductal carcinoma, compatible with no special type, originating from EBT. The patient underwent wide local excision of the lesion with clear margins, followed by adjuvant treatment with letrozole. This case highlights the importance of timely diagnosis and emphasizes the significance of recognizing the rare presentation of primary breast carcinoma in EBT. Early detection and appropriate treatment, including surgical intervention and targeted therapy, are essential for optimizing patient outcomes in such cases.

## Introduction

Ectopic breast tissue (EBT) results from incomplete regression of the embryonic mammary ridges, also known as the “milk line,” which extends bilaterally from the axilla to the groin. This developmental anomaly occurs in less than 3% of the population and may undergo physiological and pathological changes similar to normal breast tissue [1-3]. Although most EBT is benign, malignant transformation, termed ectopic breast carcinoma (EBC), is rare, representing less than 0.4% of all breast cancers [4]. Fewer than 100 cases of EBC have been documented in the literature to date, underscoring the extreme rarity of this entity [2,5].

The axilla is the most common location for EBT and EBC, likely due to its inclusion in routine breast examinations. By contrast, EBT in extra-axillary regions, such as the suprapubic area, often goes unrecognized, leading to diagnostic delays [2,3]. These lesions may mimic benign conditions such as lipomas, epidermal cysts, or postoperative granulomas. Imaging is often inconclusive due to the unusual location, making histopathology essential for diagnosis [4,5].

Immunohistochemical (IHC) analysis is crucial in confirming mammary origin and excluding metastatic or adnexal neoplasms. Tumors of mammary lineage often express markers such as GATA3, mammaglobin, and GCDFP-15 [6,7]. In addition to hormone receptor status and HER2 expression, these markers are important in differentiating EBC from cutaneous adnexal tumors or metastases from other primaries. Histologically, invasive ductal carcinoma is the most commonly reported subtype in EBC cases, typically showing features similar to its orthotopic counterpart, including glandular structures, lymphovascular invasion, and variable mitotic activity [5-7].

Previous studies, including those by Miles et al., Zhang et al., and Marshall et al., have emphasized the importance of maintaining clinical suspicion for EBC in subcutaneous nodules arising in unusual anatomical sites [1,2,5]. Despite these reports, EBC in the suprapubic region remains exceedingly rare. This case presents a primary ductal carcinoma arising in EBT within a Pfannenstiel scar, with no associated overlying nipple or skin abnormality, highlighting the diagnostic complexity and potential for misdiagnosis.

This article was previously posted to the ResearchSquare preprint server on November 26, 2023.

## Case presentation

A 69-year-old female with a family history of breast cancer, including two maternal nieces, one alive at 45 and the other diagnosed at 41, died a year after diagnosis from metastatic disease. The patient had undergone Pfannenstiel’s laparotomy for an ectopic pregnancy 43 years ago. On clinical breast examination, no palpable masses, skin retraction, or nipple discharge were observed in either breast. Axillary examination was unremarkable, with no palpable lymphadenopathy. She presented with a 2 cm subcutaneous nodule at the right end of the laparotomy scar in the prepubic region, with three years of evolution and progressive growth. On abdominal examination, the lesion appeared as a firm, mobile, non-tender subcutaneous nodule approximately 2 cm in diameter, located at the right lateral edge of the Pfannenstiel scar. The overlying skin showed no erythema, ulceration, or adherence. She consulted a surgeon who performed an excisional biopsy with a clinical diagnosis of postsurgical granuloma. The final pathology report revealed grade 2 moderately differentiated carcinoma with free margins.

Given the patient's presentation with a subcutaneous nodule in scarring tissue in the lower abdomen, which showed progressive growth but was otherwise asymptomatic, a comprehensive diagnostic approach was warranted. Despite a family history of breast cancer, which may not directly influence the diagnosis in many cases, it prompted an in-depth examination due to the unusual location. The diagnostic approach commenced with IHC analysis to identify the primary site of origin of the carcinoma. The initial pathology report following IHC analysis confirmed carcinoma of mammary origin.

**Figure 1:**
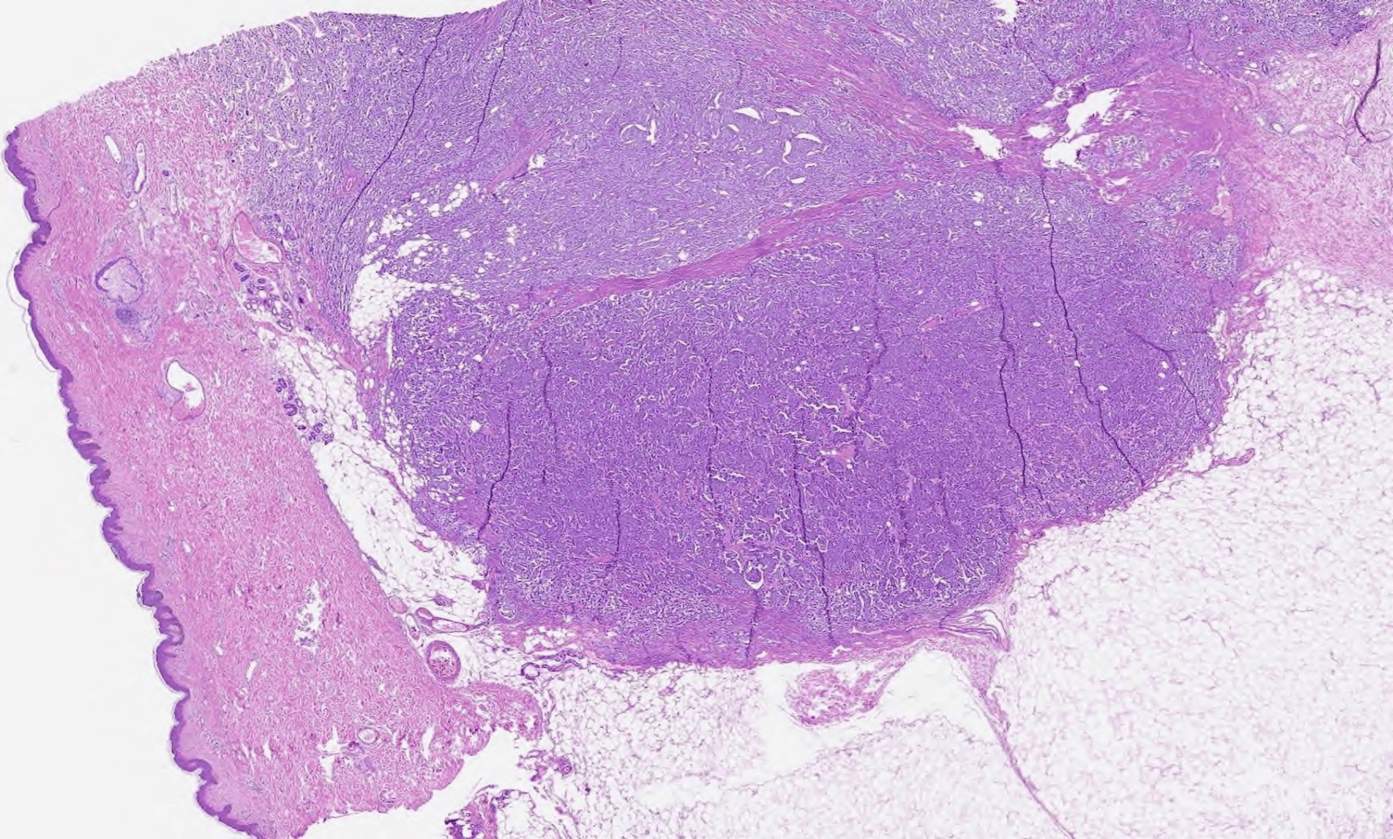
A malignant infiltrative epithelial neoplastic proliferation is identified, in the subcutaneous tissue and dermis, displaying poorly defined irregular borders. No breast tissue was identified in adjacent cutaneous appendages. The neoplasm consists of sheets, cords, and solid nests of cells with scant gland formations that diffusely infiltrate the tissue (10x).

**Figure 2:**
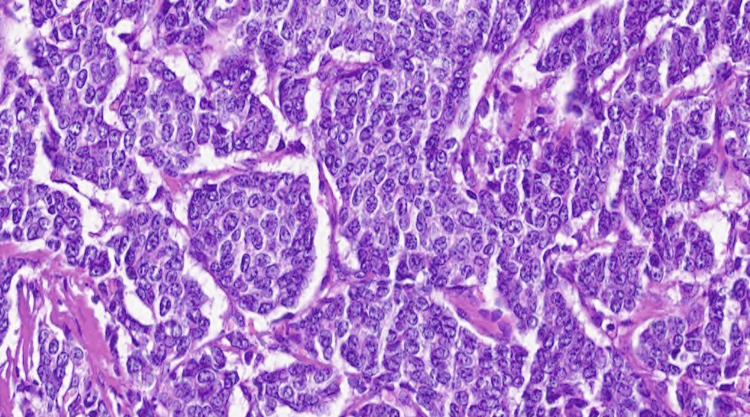
The neoplastic cells exhibit abundant pale eosinophilic cytoplasm, with enlarged, polygonal nuclei showing moderate variation in size and shape, and small visible nucleoli (400x).

**Figure 3:**
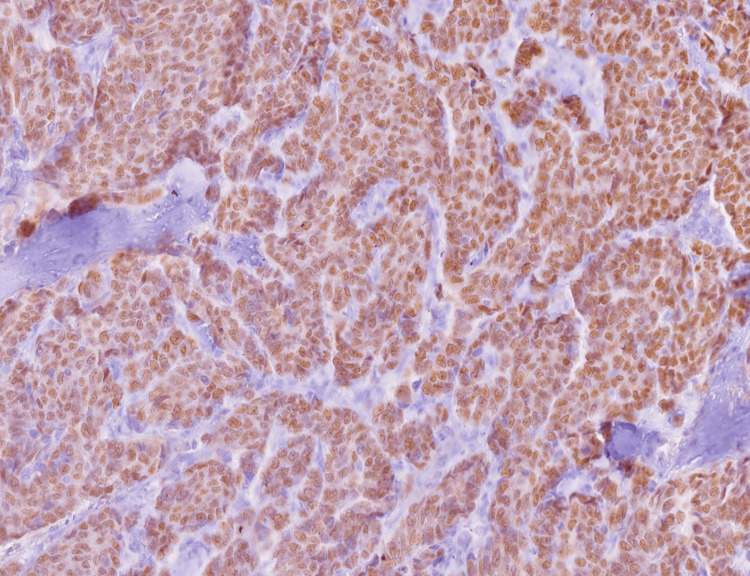
Immunohistochemical staining for estrogen receptor (ER) showing diffuse and strong nuclear positivity in neoplastic epithelial cells arranged in solid and glandular patterns, consistent with hormone receptor-positive ductal carcinoma (100x magnification).

**Figure 4:**
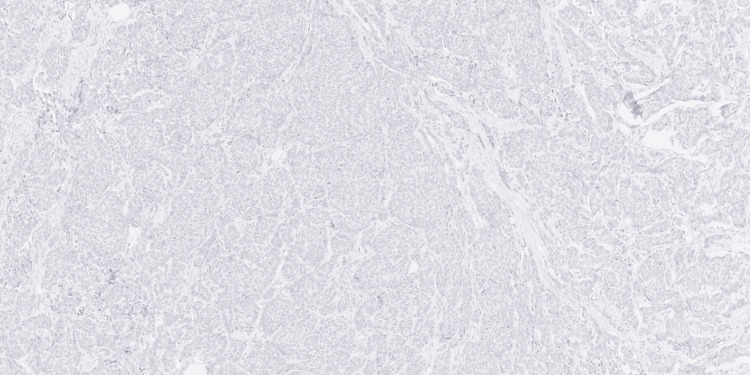
Immunohistochemical staining for HER2 showing complete absence of membranous staining in tumor cells, interpreted as HER2-negative expression (100x magnification).

Subsequent to the IHC findings, a series of radiological investigations were pursued to delineate the extent and primary origin of the malignancy. Mammography and breast ultrasound revealed a suspicious lesion in the right mammary gland, prompting further assessment due to its solid, hypoechoic, nodular characteristics reported as BI-RADS category 4. Given the absence of comparative studies, a percutaneous ultrasound-guided biopsy was undertaken, yielding a pathology report consistent with collagenized fibroadenoma. In light of inconclusive results from the initial radiological investigations, a comprehensive workup ensued, encompassing blood count, chemistry panel, abdominal computed tomography (CT) with oral and intravenous contrast, colonoscopy, and PET-CT. However, none of these studies provided definitive insights into the primary origin of the carcinoma. With diagnostic uncertainty, the patient sought a second opinion at our breast clinic 12 months later. At the Breast Care Center, Hospital Ángeles Valle Oriente, we performed bilateral mammography and breast ultrasound and reviewed the pathology slides of the excisional biopsy of the prepubic nodule. The imaging results supported the BI-RADS 2 category. Pathology findings revealed ductal adenocarcinoma of breast origin, moderately differentiated, 2 cm, with lymphovascular invasion, free surgical margins, estrogen receptors (90%), progesterone receptors (60%), and negative HER2 and Ki67 (25%) (Figures 1-4).

**Figure 5:**
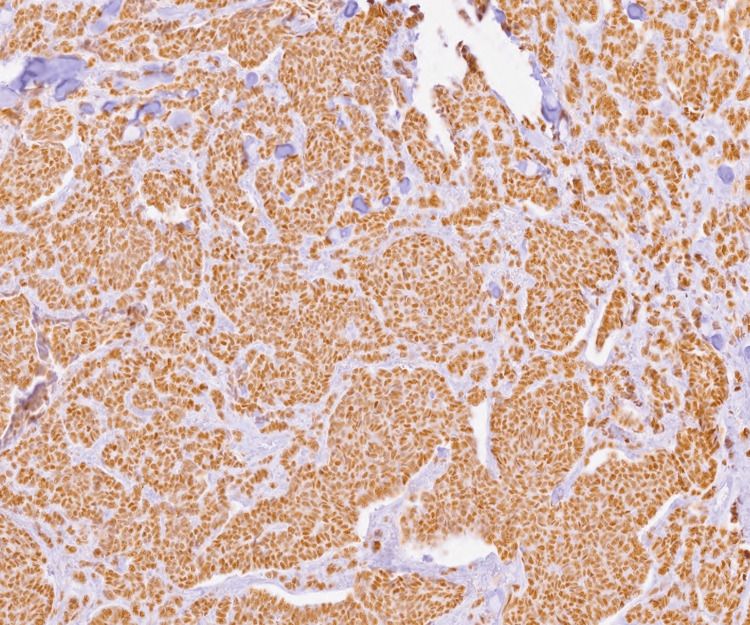
Immunohistochemical staining for GATA3 showing intense nuclear positivity in the majority of tumor cells. This finding confirms mammary epithelial origin and supports the diagnosis of ectopic breast carcinoma (100x magnification).

**Figure 6:**
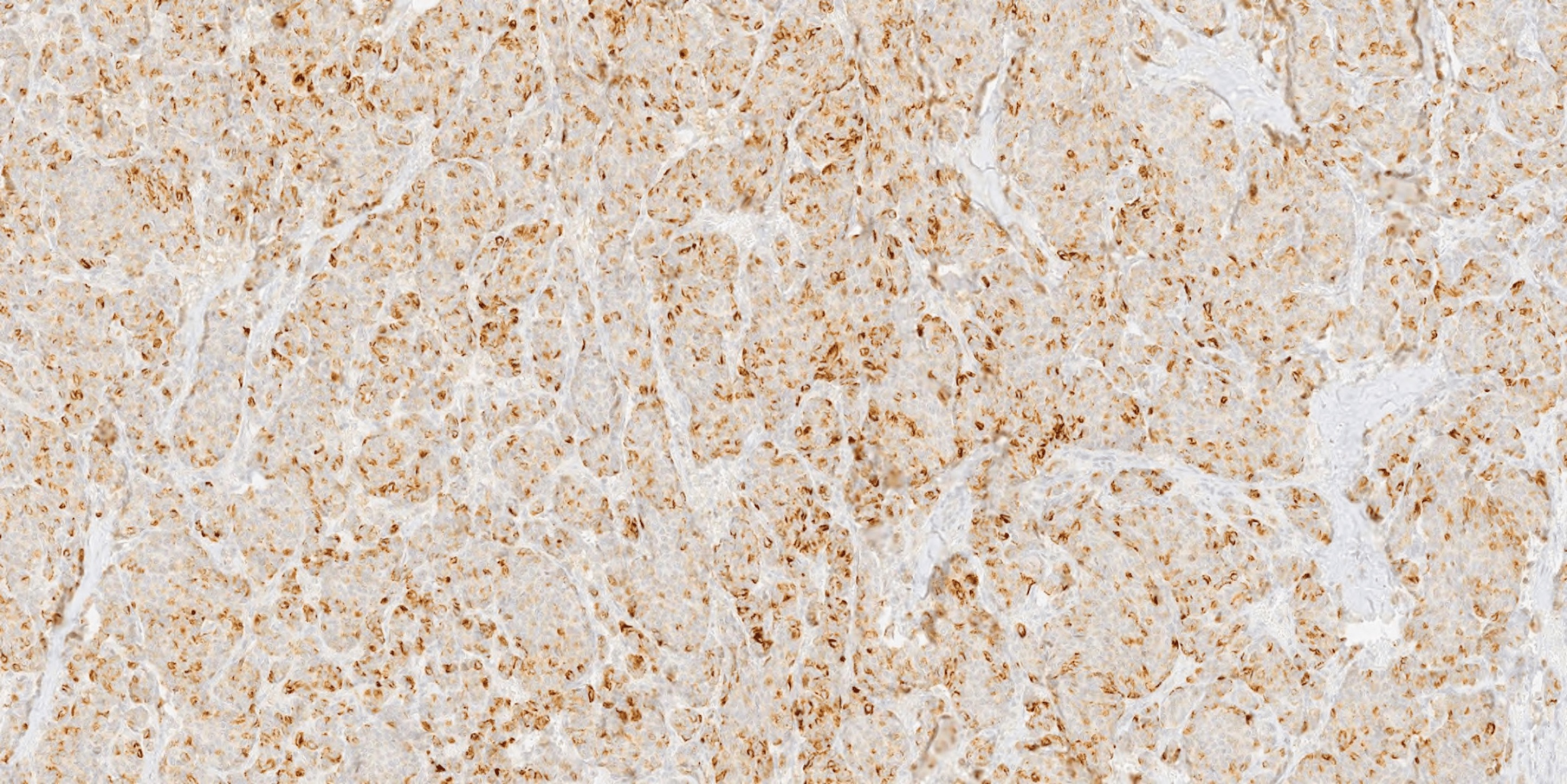
Immunohistochemical staining for GCDFP-15 showing focal cytoplasmic positivity in scattered tumor cells. GCDFP-15 expression further supports breast-specific differentiation in this ectopic mammary carcinoma (100x magnification).

In addition to the conventional immunohistochemistry panel, other markers were used to confirm the suspected presence of EBT. The results included GATA 3 (95%), mammaglobin (40%), and GCDFP15 (5%) (Figures 5-6). 

Therefore, the morphological appearance, IHC profile, absence of a lesion in the cutaneous appendages, and disease in another area, together with the clinical history, were consistent with a diagnosis of primary breast cancer from ectopic/ uninvoluted mammary tissue along the milk line in the abdominal wall.

The patient underwent adjuvant treatment and is currently stable with letrozole, disease-free, local and regional, and systemic for two years. The step-by-step approach to ruling out secondary carcinoma of breast origin and arriving at the final diagnosis underscores the challenge in diagnosing such cases and highlights the importance of comprehensive evaluation and multidisciplinary collaboration in managing patients with rare presentations of breast carcinoma.

## Discussion

Accessory breast tissue frequently develops along the embryonic mammary line or milk line, extending from the axilla to the groin due to incomplete regression, except in the anterior thoracic region, where normal breast tissue grows. This tissue may include any of the three components or a combination of nipple, areola, and parenchyma [2]. The incidence in the general population is 3%, with women accounting for over 95% of cases [1]. Similar to normal breast tissue, accessory breast tissue is susceptible to hormonal changes, and a variety of benign and malignant tumors have been reported to develop, most frequently in the axilla [3]. Primary EBC is an uncommon phenomenon, representing 0.4% of all breast cancers [1], and has been reported in only a small number of cases.

The most common clinical presentation, as seen in the majority of published case reports or retrospective studies, such as those by Zhang et al. and Famá et al., is in the axilla, occurring in 76% of cases [2-3]. Less common locations include the submammary, subclavicular, parasternal, and vulvar zones. The most affected sex is women, with a mean age at diagnosis of 54 years. However, according to Marshall et al., the age of presentation can range from 28 to 90 years [4]. A clinical manifestation often reported is a palpable mass, sometimes accompanied by edema or, less commonly, pain. Nonetheless, some patients may remain asymptomatic, further delaying diagnosis. As with conventional breast cancer, mammography and ultrasound can support early detection; however, definitive diagnosis relies on histopathology. In cases of EBC, IHC markers such as GATA3, mammaglobin, and GCDFP-15 play a critical role in confirming mammary origin and distinguishing these tumors from adnexal or metastatic neoplasms [6-7]. The most commonly reported histological subtype, accounting for over 76% of cases, is invasive ductal carcinoma [5].

Our patient’s presentation was unusual. She had a symptomatic subcutaneous nodule in the suprapubic region at the end of a laparotomy scar, which can easily be mistaken for skin lesions or postoperative granuloma. As reported in similar cases, a delay in diagnosis occurred because EBC was not initially suspected. Unlike the majority of literature, where axillary presentation predominates, our patient’s lesion was located in an extra-axillary zone. In our review, the only abdominal case previously reported involved a supernumerary nipple with surrounding erythema, which could raise suspicion of EBT. However, that was not the case in our patient, complicating the diagnostic approach.

Treatment for EBC parallels that of breast cancer in its usual anatomical location. It includes surgery, chemotherapy, radiotherapy, and endocrine therapy. Standard care involves complete tumor excision and may also require adjuvant systemic therapy and radiation for local control, particularly in advanced or high-risk cases. For patients with a prior history of breast cancer, radiation may be considered if the ectopic lesion lies outside previously treated fields [1].

## Conclusions

Ectopic breast cancer is a rare but clinically significant differential diagnosis for subcutaneous nodules in non-traditional locations along the embryologic milk line. Its low prevalence and the absence of anatomical breast parenchyma often lead to misdiagnosis, unnecessary interventions, and delays in appropriate care. This case reinforces the importance of maintaining a high index of suspicion when evaluating unexplained soft tissue masses, especially in extra-axillary regions, where clinical suspicion may be low. Diagnosis relies on a multidisciplinary approach, integrating detailed histopathological analysis and immunohistochemical profiling to confirm mammary origin. These tools are essential for distinguishing EBC from other adnexal or metastatic neoplasms. Although the therapeutic strategies generally follow standard breast cancer protocols, timely recognition is key to avoiding mismanagement and ensuring optimal outcomes. Thorough physical examination, including abdominal assessment, plays a key role in detecting these unusual presentations and guiding further diagnostic steps, especially when imaging is inconclusive or when nodules arise in surgical scars or unusual locations. By raising awareness of these atypical presentations among clinicians, pathologists, and surgeons, we can improve diagnostic accuracy, accelerate treatment decisions, and ultimately enhance patient prognosis and quality of care.
